# Burn Injury: Mechanisms of Keratinocyte Cell Death

**DOI:** 10.3390/medsci9030051

**Published:** 2021-07-16

**Authors:** Hans-Oliver Rennekampff, Ziyad Alharbi

**Affiliations:** 1 Department of Plastic Surgery, Hand and Burn Surgery, Burn Center, Rhein Maas Klinikum, 52146 Wuerselen, Germany; 2 Plastic Surgery and Burn Unit, Fakeeh Care & Fakeeh College of Medical Sciences, P.O. Box 2537, Jeddah 21461, Saudi Arabia; zialharbi@fcms.edu.sa

**Keywords:** burn, wound, keratinocyte, apoptosis, cell death, review

## Abstract

Cutaneous burn injury is associated with epidermal loss in the zone of coagulation zone and delayed tissue loss in the zone of stasis. Thus, thermal stress can trigger both necrosis and regulated cell death (RCD) or apoptosis. Experimental in vitro and in vivo work has clearly demonstrated apoptotic events of thermally injured keratinocytes that are accompanied by morphological and biochemical markers of regulated cell death. However, in vivo data for the different pathways of regulated cell death are sparse. In vitro experiments with heat-stressed human keratinocytes have demonstrated death receptor involvement (extrinsic apoptosis), calcium influx, and disruption of mitochondrial membrane potential (intrinsic apoptosis) in regulated cell death. In addition, caspase-independent pathways have been suggested in regulated cell death. Keratinocyte heat stress leads to reduced proliferation, possibly as a result of reduced keratinocyte adhesion (anoikis) or oncogene involvement. Understanding the underlying mechanisms of RCD and the skin’s responses to thermal stress may lead to improved strategies for treating cutaneous burn trauma.

## 1. Introduction

Cutaneous burn injury is associated with epidermal loss that necessitates skin grafting in deep partial and full-thickness burns. Studies by Moritz and Henriques have analysed thermal stress to the skin and have demonstrated that the death of cells is time and temperature dependent [1]. In 1953, Jackson reported that burn wounds were typically accompanied by necrosis in the zone of coagulation and delayed tissue loss in the zone of stasis [2]. While high temperatures may cause immediate cell death in cutaneous tissue, referred to as accidental cell death (ACD), sublethal heat present in the zone of stasis may lead to the destruction of keratinocytes through regulated cell death (RCD) [3]. Recent publications have identified regulated cell death in cutaneous burn injury [4,5,6,7]; however, little is known about the biological mechanisms involved in heat stress-induced apoptosis of the skin.

Cell death can be identified through biochemical or histopathological changes. Three different forms of histopathological alterations have to be distinguished: (1) type I cell death or apoptosis exhibiting cytoplasmic shrinkage, chromatin condensation (pyknosis), nuclear fragmentation (karyorrhexis), and plasma membrane blebbing; (2) type II cell death or autophagy manifesting with extensive cytoplasmic vacuolisation and phagocytic uptake and consequent lysosomal degradation; (3) type III cell death or necrosis with no distinctive features of type I or II cell death [3,8].

Further biochemical and functional research has deciphered the molecular basis of RCD, which has resulted in a more differentiated nomenclature [3]. In addition to the classic pathway distinction between death receptor-initiated extrinsic apoptosis and intrinsic apoptosis, caspase-dependent and caspase-independent intrinsic apoptosis must also be distinguished. The following pathways for RCD have been described: intrinsic apoptosis, extrinsic apoptosis, mitochondrial permeability transition (MPT)-driven necrosis, necroptosis, ferroptosis, pyroptosis, parthanatos, entotic cell death, NETotic cell death, lysosome-dependent cell death, autophagy-dependent cell death, immunogenic cell death, cellular senescence, and mitotic catastrophe. A review of the literature revealed that some, but not all, of these RCDs have been identified for keratinocyte death [8].

Molecular features of apoptosis such as activation of endonucleases and degradation of DNA were also identified during terminal differentiation of keratinocytes in the epidermis [9]. While terminal differentiation generates functionally important cells [10], stress-induced apoptosis causes the selective deletion of individual cells. Cellular senescence of the epidermis shares some apoptotic characteristics such as expression of Fas and Fas ligand below the granular layer of the epidermis [11]. However, growth-arrested, senescent keratinocytes become resistant to apoptosis by inactivating the function of p53 [12]. In summary, it seems useful to distinguish stress-induced apoptosis from terminal differentiation and senescence in the skin.

Specifically, this review will focus on the impact of in vitro and in vivo thermal stress on keratinocyte death at the functional, molecular, and histopathological levels. Possible therapeutic interventions which may improve burn wound healing will be discussed. Understanding the underlying mechanisms and responses of the skin to thermal stress may lead to improved strategies for treating burn trauma.

## 2. Accidental Cell Death (ACD)

Physical contact with extremely high temperatures instantaneously results in accidental cell death with physical disassembly of the plasma membrane [13]. Necrotic cells should be distinguished from apoptotic cells or autophagy by the absence of pyknosis, karyorrhexis, cytoplasmic vacuolisation, and lysosomal degradation. In 1947, Moritz and Henriques meticulously analysed the influence of time and temperature on epidermal necrosis [1]. In their set of experiments, applying a temperature of over 70 °C for a single second led to necrosis of the epidermis. Matylevitch et al. used in vitro staining with calcein AM and ethidium bromide to detect necrotic keratinocytes after thermal injury [14]. Similar to the above-mentioned in vivo experiments by Moritz and Henriques, a temperature of 72 °C was applied for one second, which induced necrosis in cultured keratinocytes.

## 3. Apoptosis of Keratinocytes In Vitro

**Figure 1:**
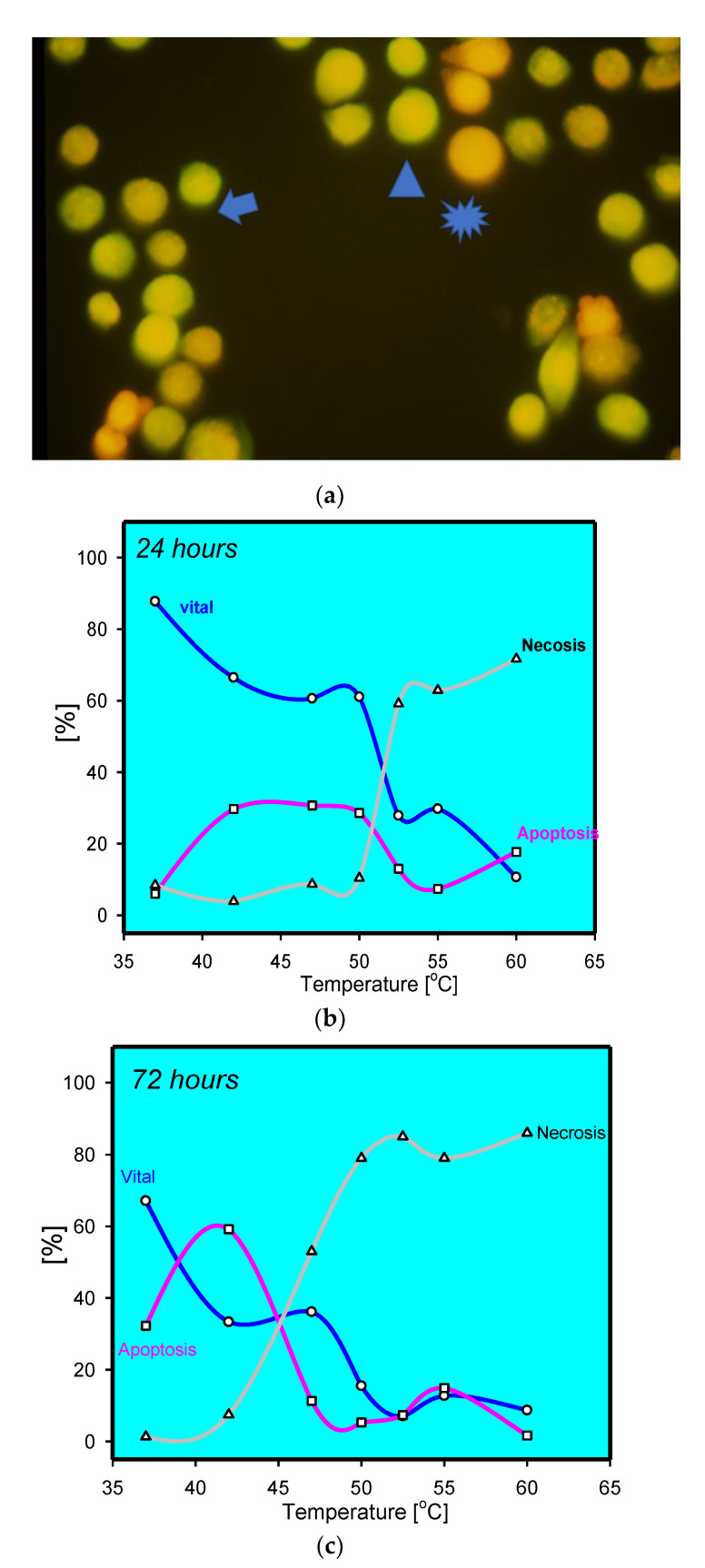
(**a**) Staining of thermally stress keratinocytes at 47 °C after 72 h. Viable cells have intense green fluorescence (arrow head). Apoptotic cells with reduced green fluorescence and condensed red fluorescence (arrow). Homogenous red fluorescence indicative for necrotic cells (star); (**b**) percentage of live, apoptotic, and dead keratinocytes after thermal stress for 5 min at the given temperature analysed by acridine orange/ethidiumbromide, indicating a sharp increase in necrotic cells above 50 °C and decrease in viability. Apoptotic keratinocytes were mainly observed at moderate temperatures; (**c**) percentage of live, apoptotic, and dead keratinocytes after thermal stress for 5 min at the given temperature analysed by acridine orange/ethidiumbromide after 72 h; notice the increase in apoptotic keratinocytes at moderate temperatures.

A variety of in vitro studies have evaluated the impact of heat on keratinocytes. The experiments involved HaCaT cells as well as primary keratinocytes. However, HaCaT cells resemble a spontaneously immortalised human keratinocyte cell line calling into question regular apoptotic pathways [15]. It is worth mentioning that cultured keratinocytes may only resemble basal proliferative keratinocytes, while the epidermis is a complex stratified squamous, keratinised epithelium consisting of basal, spinous, granular, and cornified cell layers. In most, if not all, experiments, cultured primary keratinocytes heated to 60 °C—generally, a critical temperature in vitro—for various lengths of time were analysed for apoptotic events. Apoptosis was morphologically determined on the ultrastructural level using electron microscopy with cell shrinkage, cytoplasmatic budding, and alterations of nuclear morphology [14]. In addition, DNA laddering, another classic feature used to establish apoptosis, was detected following heat stress in primary keratinocytes. Histologically, the free ends of fragmented DNA can be labelled using TdT-mediated dUTP biotin nick end-labelling (TUNEL) in thermally stressed keratinocytes in vivo (Supplementary Materials Figure S1) [4,5,6,7]. Analysing time-dependent alterations in keratinocytes after heat stress using acridine orange/ethidium bromide revealed an increase of apoptotic/dying cells over time, similar to the clinically observed delayed tissue loss (Figure 1) [16].

### 3.1. Intrinsic Apoptosis

**Figure 2:**
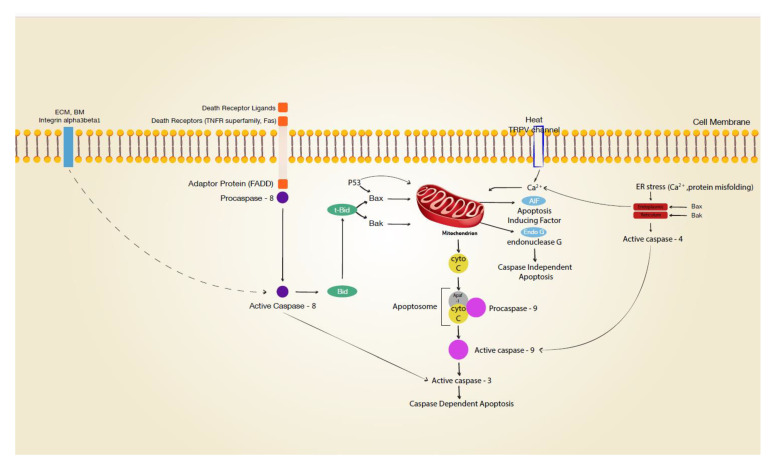
Signal transduction leading to apoptosis. Various cellular stress, e.g., matrix disruption, death receptor activation, transient vanilloid receptor (TRPV) opening, protein misfolding may initiate apoptotic pathways in keratinocytes. ECM extracellular matrix; BM basement membrane; dotted line indicating activation after integrin internalisation.

Mitochondria play a central role in intrinsic apoptosis (Figure 2) [3]. Mitochondrial dysfunction as a hallmark of apoptosis is accompanied by decreased membrane potential (ΔΨm). It has been reported that an intracytoplasmic rise in Ca^2+^ depolarises the inner membrane of mitochondria, with a subsequent release of cytochrome c, formation of the apoptosome (with PARP-1), and cleavage of procaspase-9 to caspase 9 [17,18]. Heat-sensitive transient receptor potential vanilloid channels (TRPV1 and TRPV2) are expressed on human keratinocytes and can be stimulated by high temperatures (>43 °C and >52 °C, respectively) [19,20]. Opening these ion channels results in an intracellular calcium rise [21], which may well affect mitochondrial function. Positive staining for mitochondrial membrane potential disruption in thermally stressed keratinocytes in vitro suggested critical involvement of the mitochondrium in cutaneous burn injury [20]. However, interpretation of this finding is complicated by the fact that different regulated cell death pathways involving mitochondria have to be distinguished, such as the intrinsic pathway with cytochrome c release, the AIF/Endo G pathway, the lysosomal pathway, and MPT-driven necrosis [3]. Neither involvement of peptidylprolyl isomerase F (Cyclophilin D, CYPD), which is specifically linked to mitochondrial permeability transition (MPT)-driven necrosis, nor the lysosomal pathway via cathepsin have been examined for cutaneous thermal injury. Yet, heat-stress-induced cathepsin B release from lysosomes with downstream activation of caspase 9 was reported for cells from the small intestine [22]. In contrast, Chinnathambi et al. [23] reported that apoptosis of thermally injured keratinocytes occurs via caspase-independent AIF/EndoG pathways. The latter pathway is linked to PARP1 activation (parthanatos), with possible significance for wound healing [24,25]. Cell stress, such as UV irradiation, can result in mitochondrial release of cytochrome c and APAF 1, which recruit procaspase 9, forming the apoptosome and subsequently activating caspase 9 [26]. While the involvement of the initiator caspase 9 has not been studied in thermal injury, Holmes et al. were able to detect the executioner caspase 3 after thermal stress in vitro, indicating a caspase-dependent pathway in thermally injured keratinocytes [27].

Dysregulation of mitochondria was reported for two other proapoptotic modulators, Bak and Bax [17,18], both of which are expressed in keratinocytes [8,28]. These proteins are linked to the mitochondrium, and upon activation by truncated Bid (tBid), they are critically involved in mitochondrial outer membrane permeabilisation. It is of note that it was reported that the tumour suppressor gene p53 can directly activate Bax [29]. Other studies have suggested that p53 directly translocates to the mitochondrium [30,31,32,33]. In previous research [34], we demonstrated p53 phosphorylation and stabilisation immediately after heat stress in human keratinocytes. While it has been linked to heat-induced apoptosis in testis and cell lines [32,33,35], the precise involvement of Bax (with or without p53 translocation) in the thermal injury of the skin remains unknown. Notably, activation of Bak and Bax has also been discussed in permeabilisation of the endoplasmic reticulum with subsequent Ca^2+^ release and apoptosis (see below) [36].

Research on hyperthermia for cancer treatment has indicated that the endoplasmic reticulum (ER) is involved in heat-induced apoptosis [37,38]. Loss of cellular homeostasis or calcium influx into the cell disrupts Ca^2+^ signaling, inducing an ER stress response [39,40]. Downstream events of ER stress include (but are not limited to) caspase 4 activation (human ortholog of caspase 12), which leads to cleavage of caspase 3. It has been reported that cells can also apoptose without ER-associated caspases 4 and 12 in a different caspase-dependent pathway [41,42].

### 3.2. Death-Receptor-Linked Apoptosis

Death-receptor-linked apoptosis is initiated by signals from the extracellular environment detected by cell membrane receptors (death receptors) and is propagated downstream via the adaptor Fas-associated death domain (FADD) to activate the initiator caspase 8 [8,43]. In turn, this extrinsic pathway is either connected with the intrinsic pathway via caspase-8-dependent Bid truncation or may lead to cleavage of the executioner caspase 3. Death receptors found on keratinocytes are the Fas cell surface death receptor (Fas—also known as CD95 or APO-1) and TNF receptor superfamily members 1A (TNFR1), 10a (TRAILR1 or DR4), and 10b (TRAILR2 or DR5) [8]. Death receptor ligands (sFasL/CD95L, TNF-α, and TRAIL) are mainly expressed by immune cells but are also released by keratinocytes [8]. Whether suicidal death occurs in burn wounds must be determined in light of the concentration of these molecules in blister fluids and the concentrations required to induce apoptosis. The mean concentration of TRAIL in burn blister fluid was 316 pg/mL [44], TNF-α ranged from 0 to 3 pg/mL with a mean of 0.96 pg/mL [45], and sFasL measured 18 pg/mL [46]. Human recombinant sFas-L induced apoptosis of keratinocytes has been demonstrated with concentrations from 89 to 232 ng per mL [47]. The administration of 125 ng to 250 ng per mL TRAIL induced an apoptotic cell death rate of 30% in cultured keratinocytes [48]. Given the reported concentrations of death receptor ligands, it is questionable whether they are clinically involved in apoptotic cell death in burns. Active caspase 8, the initiator caspase involved in signal transmission from death receptors, has been demonstrated in thermally stressed keratinocytes in vitro. However, initiator caspase 8 activation does not necessarily indicate death receptor involvement, as caspase 8 can also be activated by ER stress [39] or by ß1-integrin blockage [49].

### 3.3. Apoptosis of the Skin

While in vitro experiments may allow different pathways of apoptotic cell death to be analysed, histology from thermally injured skin is descriptive of post-burn events that are actually occurring. However, there is substantial evidence that apoptosis or regulated cell death occurs in post-burn skin. Still, reports on in vivo events after thermal trauma are sparse. Immunohistological detection of cleaved caspase 3 (Figure S2) [7,27] has revealed a caspase-dependent pathway in the thermally injured epidermis but may not exclude an additional caspase-independent pathway, such as an AIF/EndoG pathway. Expression of the executioner caspase 3 calls for upstream activation of initiator caspases 8, 9, or 4. Immunoreactivity for caspase 8 was histologically confirmed [7], suggesting a death receptor pathway in human skin after thermal trauma. Subsequent histology revealed FAS immunoreactivity and TNFR1 immunoreactivity on keratinocytes at the edges of burn wounds. Whether the expression of death receptors and the concentrations of their ligands are sufficient to induce apoptosis remains an open question (see the section on death-receptor-linked apoptosis). While caspase 9 immunoreactivity has not been analysed in vivo after thermal stress, an intrinsic pathway has not been excluded, as Bax was identified on the mRNA level in murine skin after thermal trauma [50]. However, the precise involvement of mitochondria or the endoplasmic reticulum (ER) in thermally induced regulated cell death has not been analysed in human skin. With heat-sensitive TRPV channel immunoreactivity reported on keratinocytes in vivo [19,20] and heat-induced Ca^2+^ influx in vitro [21], either an ER or mitochondrial pathway seems possible after heat stress. While Chinnathambi et al. [23] excluded APAF1 and cytochrome c (apoptosome)-dependent mitochondrial pathways, others [51] demonstrated APAF1 immunoreactivity in vivo, which was indicative of a caspase-dependent pathway.

Reddy et al. [52] reported a pathway of regulated cell necrosis (necroptosis) in burn injury that involved death-receptor-mediated activation of RIPK3 (receptor-interacting serine/threonine–protein kinase 3). RIP kinases (RIPK1/RIPK3, necrosome) are critically involved in this caspase-independent pathway of regulated necrosis, which can be induced through a death receptor-dependent upstream pathway, leading to cell lysis and the release of immunogens [53,54]. In contrast to other diseases, such as cerebrovascular disease [55] and ischemia/reperfusion injury in skin flaps [56], blocking this pathway did not reduce cell death in thermally injured skin, which calls into question the importance of necroptosis in burns. Given that caspase 8 inhibits this necrotic pathway [57] and immunoreactivity of caspase 8 detectable in burned tissue, an examination of the precise role of necroptosis in cutaneous burn injury is required.

### 3.4. Inhibition of Keratinocyte Proliferation

Anoikis is a specific variant of apoptosis initiated by the loss of integrin-dependent anchorage [58,59]. It is well established that the proliferation and growth of keratinocytes in vitro is dependent on integrin-mediated adhesion to a substrate. Consequently, cell detachment from an extracellular matrix, especially the basement membrane with disruption of the α3β1 integrin, activates this apoptotic pathway [60,61]. Downstream from this event, caspase 8 and caspase 3 are activated with subsequent apoptosis [62]. It can be hypothesised that thermally induced loss of function of α3β1 integrins may exacerbate keratinocyte cell death in superficial partial-thickness burn wounds over time, leading to progression of the injury. Additionally, degradation of the extracellular matrix by thermal energy [63] or cleavage by molecules such as granzyme B [64] may contribute to loss of anchorage in the skin with subsequent anoikis. Interestingly, heat shock induces matrix metalloproteinase expression that will further degrade matrix molecules necessary for keratinocyte anchorage [65]. While thermally stressed keratinocytes in a culture show a marked decrease in proliferation, further proof of anoikis in thermally damaged skin in vivo has yet to be established.

## 4. Therapeutic Outlook

The ultimate goal of understanding the mechanisms of cell death is to prevent tissue injury in pathophysiologic processes resulting from events such as thermal injury to the skin. A common question, therefore, is which form of cell death—apoptosis, necrosis, or a mixture—is occurring in this disease process. Currently, there are only a few immediate (on the scene) therapeutic interventions that are administered clinically to reduce heat-induced cell damage. Interestingly, these include cooling as well as warming [66]. In vitro, we could neither see improved cell survival nor proliferation with immediate cooling with 15 °C cold fluid [34]. A variety of pharmacological interventions, as well as cell-based applications, have been reported to ameliorate apoptosis in cutaneous thermal injury (Table 1). At present, no drug has clearance by the FDA for the treatment of burn-induced apoptosis. Some of the reported therapies have dual functions acting via growth-factor-induced proliferation and, at the same time, reducing apoptosis shown by diminished TUNEL positive cells or caspase 3 activity. Attenuating oxidative stress by systemic drugs such as methylene blue or Astaxanthin is another therapeutic approach [67,68]. Most studies have used in vitro models or animal models, often with a preburn therapeutic intervention, which calls into question their clinical usefulness. The ideal timing for postburn therapeutic administration is also challenging because the exact point of no return for apoptosis in thermally injured keratinocytes has yet to be determined.

## 5. Conclusions

Understanding the mechanisms of cell death in cutaneous burn injury is a prerequisite for preserving keratinocytes of the epidermis and subsequently preventing sequelae, such as skin grafting or scarring. It is now well established that different modes of cell death occur after thermal injury to keratinocytes in vitro. Evidence from various groups indicates an intrinsic pathway involving mitochondria with downstream caspase-dependent apoptosis. However, there is still sparse information on whether these mechanisms of regulated cell death are of clinically significant relevance. Descriptive histological studies have proposed both caspase-dependent and caspase-independent pathways. Further in vivo proof-of-concept studies on blocking different pathways are warranted to decipher the precise pathophysiological relevance with functional and morphologic endpoints. In addition, further analyses of isolated keratinocytes are needed to delineate the signaling in order to determine the point of no return and timing of postburn interventions.

## Figures and Tables

**Table 1:** Postburn interventions aimed at reducing thermally induced apoptosis.

Intervention	Effect	Burn Model	Timing	Reference
Human amniotic MSC	Decreased TUNEL staining	mouse	postburn	Li et al. [69]
Inhibitor of c-jun	Decreased cell death	mouse	potburn	Giles et al. [70]
Rapamycin	Decreased TUNEL staining	rat	postburn	Xiao et al. [71]
Methylene Blue	Reduced necrosis	rat	postburn	Rosique et al. [67]
Granzyme B inhibitor	Improved healing	mouse	postburn	Shen et al. [64]
Hsp90α fragment F-5	Reduced caspase 3 staining	pig	postburn	Bhatia et al. [72]
Platelet Rich Plasma	Reduced TUNEL	rabbits	postburn	Uraloğlu et al. [73]
Astaxanthin	Reduced TUNEL staining, reduced cytochrome C and caspase 9		postburn	Fang et al. [68]
Melatonin	Reduced caspase 3 staining	rat	postburn	Zhang et al. [74]
p38MAPK inhibition	Reduced TUNEL	rat	postburn	Ipaktchi et al. [75]

MSC-mesenchymal stem cells, TUNEL TdT-mediated dUTP biotin nick end-labelling.

## Supplementary Materials

The following are available online at https://www.mdpi.com/article/10.3390/medsci9030051/s1, Figure S1: TUNEL staining of nuclei at the wound edge of a burn wound; Figure S2: Active Caspase 3 immunoreactivity is seen in keratinocytes and some dermal cells in human burn wounds.
