# Supplementary material for: Burn Injury: Mechanisms of Keratinocyte Cell Death
Source: Med Sci (Basel). 2021 Jul 16;9(3):51. doi: 10.3390/medsci9030051 (PMC8293431; doi:10.3390/medsci9030051)
Supplement: Supplementary file 1 [file medsci-09-00051-s001.zip › medsci-1251333-supplementary.pdf]

medical sciences

MDPI

Review

Burn Injury: Mechanisms of Keratinocyte Cell Death-Supplement

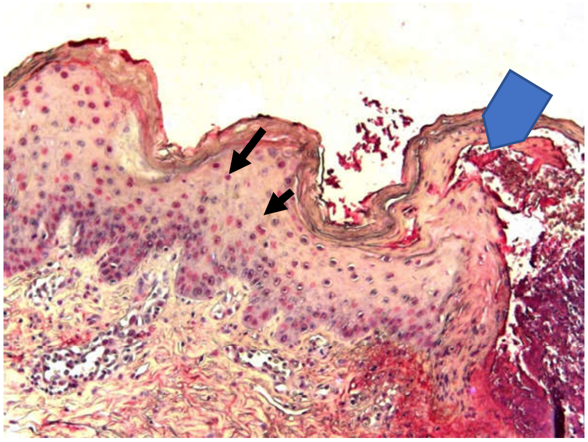

Figure S1. TUNEL staining of nuclei (arrows, red colour) at the wound edge of a burn wound; wound margin (bold arrow) his is a figure. Schemes follow another format.

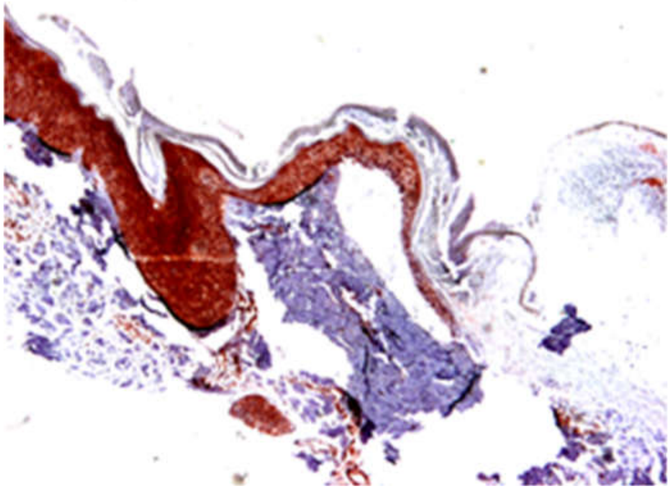

Figure S2. Strong active Caspase 3 immunoreactivity (red staining) is seen in keratinocytes and some dermal cells in human burn wounds.
